# Human retinal organoids for modelling dry age-related macular degeneration and screening drugs

**DOI:** 10.1016/j.gendis.2025.101593

**Published:** 2025-03-07

**Authors:** Xiaolu Hao, Lu Du, Guang Liu, Zhaohui Li, Shaojun Wang

**Affiliations:** a Senior Department of Ophthalmology, The Third Medical Center of Chinese PLA General Hospital, Beijing 100039, China; b Medical School of the Chinese PLA General Hospital, Beijing 100039, China; c State Key Laboratory of Common Mechanism Research for Major Diseases, Institute of Basic Medical Sciences, Chinese Academy of Medical Sciences & Peking Union Medical College, Beijing 100005, China

**Keywords:** Dry age-related macular degeneration (AMD), Fetal hemoglobin inducer TN1, Metformin, Oxidative stress, Retinal organoid

## Abstract

Age-related macular degeneration (AMD) poses a significant threat to the vision of the elderly population globally. Unfortunately, there is no effective treatment available for dry AMD. In this study, we utilized human retinal organoids (ROs) stimulated with sodium iodate to establish a model for dry AMD. We assessed the apoptosis of retinal organoid cells and conducted RNA sequencing to analyze molecular changes. Our findings indicate that metformin and the fetal hemoglobin (HbF) inducer TN1 could protect ROs from sodium iodate induced damage and restore retinal function in murine model. The administration of metformin and TN1 alleviated apoptosis in ROs and improved visual function. Studies of molecular mechanisms indicated that the protective effects of metformin and TN1 may be related to the HMOX1 gene, providing valuable insights for the development of new therapies for dry AMD via targeting HMOX1 and its downstream pathways.

## Introduction

Age-related macular degeneration (AMD), classified into dry and wet types, is a serious disease that can cause blindness among elderly individuals worldwide, and no effective medicine for dry AMD has been developed.^1^^,^^2^ The significant change in AMD is the formation of subretinal drusen in the macular region, followed by the progressive degeneration of retinal pigment epithelial cells (RPEs) and photoreceptor cells (PCs).^3^ Clinical observations, epidemiological investigations, and clinical pathology research have demonstrated that AMD may be caused by the disruption of normal retinal homeostasis mechanisms caused by aging, oxidative stress, and chronic inflammation, leading to damage to RPEs and PCs.^4^^,^^5^ Despite some progress in animal models,^6^ the exact molecular mechanisms underlying the injury of RPEs and PCs remain unclear,^7^ and little advantage has been made in the development of human retinal models that can mimic dry AMD.^8^^,^^9^ This limitation impedes further development and application of novel therapeutic strategies of dry AMD.

By using specific small-molecule compounds, human pluripotent stem cells (hPSCs) can be induced to form various organoids resembling tissues like the retina, heart, brain, thymus, intestine and liver.^10^, ^11^, ^12^ These organoids emulate human tissue structures and functions, thereby aiding in research on organ development and disease mechanisms.^13^ Single-cell RNA sequencing (scRNA-seq) analysis has demonstrated that retinal organoids (ROs) derived from hPSCs effectively mimic human retinal structure.^14^, ^15^, ^16^, ^17^, ^18^ Our previous work indicated that ROs contain abundant RPEs and PCs at 186 days of differentiation,^19^ underscoring their potential as model for human retinal diseases and the development of novel therapeutic strategies.^20^ Therefore, these ROs have greatly advanced our understanding of human retinal development^21^ and have been used to reveal the pathological processes and related molecular mechanisms underlying retinal dysfunction caused by genetic mutations in recent years.^22^, ^23^, ^24^

Consequently, we speculate that ROs show great promise in elucidating the pathogenesis of AMD and in identifying new therapeutic targets. Prior research has indicated that the systemic administration of sodium iodate can effectively simulate the key process of oxidative stress damage observed in dry AMD.^25^ Therefore, in this study, we propose to develop an in vitro AMD model (hRO-AMD) by incorporating oxidative stress inducers into ROs. Furthermore, we aimed to explore the mechanism related to retinal injury and assess the protective effects of metformin and the fetal hemoglobin (HbF) inducer TN1, with the hope of contributing to the development of innovative therapies for AMD.

## Materials and methods

### Culture of human embryonic stem cells

The human embryonic stem cell line (hESC, H9, Passage 61st) was kindly provided by Professor Huang Yue (Department of Medical Genetics, Institute of Basic Medical Sciences, Chinese Academy of Medical Sciences & Peking Union Medical College). After mycoplasma contamination was excluded, the hESC line was maintained in E8 culture medium (Thermo Fisher, Cat. A1517001) on VTN-coated dishes under 5% CO_2_ conditions. For passaging, after aspiration of the culture medium, the cells were washed twice with DPBS (Life Technology, Cat. 14190144), incubated with Versene (Life Technology, Cat. 15040066) at 37 °C for 5 min, broken into smaller pieces by gentle pipetting, and resuspended in E8 culture medium with 10 mM ROCK inhibitor Y27632 (R&D, Cat. 1254). Twenty-four hours later, the medium was replaced with culture medium without Y27632. The passages were performed at a 1:6 split ratio every 3rd day. After 6 passages, the hESCs were tested for core transcripts, and three-dimensional (3D) ROs differentiation was started.

### 3D differentiation of retinal organoids

A previously reported protocol was adopted.^19^ When the cells reached 70% confluence, the hESCs were digested into a single-cell suspension with TrypLE+10 mM ROCK inhibitor Y27632 (ThermoFisher, Cat 12604013). After centrifugation (300 *g* ∗ 5min) to remove the digestion solution, gfCDM (supplemented with 10% KSR, 45% Discover's modified Dulbecco's medium (Gibco, Cat. 10828028, 11965118), 45% Hams F12 (Gibco, Cat. 11765054), GlutaMAX (1% chemically defined lipid concentration (Gibco, Cat. 11905031), 10 mM ROCK inhibitor Y27632 (ThermoFisher, Cat 12604013) and 450 mM monothioglycerol (Sigma, Cat. M6145) were used for resuspension by plating 10,000 cells per well into a 96-well plate at the bottom of the V- bottom type (Sbio, Cat. MS-9096MZ). On the 6th day, the medium was replaced with fresh medium containing BMP4 (1.5 nM, Peprotech, Cat. AF-120-05ET), and half of the medium was replaced every 3rd day. At the beginning of the 18th day, the ROs were transferred to a low-adsorption culture dish (30 ROs per dish). The medium was replaced with DMEM/F12-GlutaMAX medium supplemented with 1% N2, 3 mM CHIR99021 (Sigma, Cat. 252917-06-9), and 5 mM SU5402 (Sigma, Cat. SML0443). On the 24th day, DMEM/F12-GlutaMAX medium (Gibco, Cat. 10565-018), 1% N2 supplement (Gibco, Cat. 17502-048), 10% KSR, 0.5 mM retinoic acid (Sigma, Cat.R2625), and 0.1 mM taurine (Sigma, Cat. T0625) were added. After 90 days of differentiation, retinoic acid (RA) was removed from the medium for long-term culture differentiation.

### Protocols for the retinal degeneration model

For the degeneration protocols of ROs, ROs were placed into Petri dishes with different concentrations of sodium iodate (NaIO_3_) (0.1, 1, or 10 mM, Sigma, Cat. 7681-55-2). The medium supplemented with sodium iodate was changed every 3rd day during the experiment.

### Drug application

For retinal degeneration drug testing, metformin (Selleck, Cat. S5958, USA) and the HbF inducer TN1 (MedChemExpress, HY-100826, USA) were dissolved according to the manufacturer's instructions. ROs were pretreated with 100 mM metformin or 100 mM TN1 for 7 days before NaIO_3_ application.

### Immunofluorescence staining

For immunostaining, the sections were dewaxed, subjected to antigen retrieval and the coverslip of cells samples were blocked with 10% goat serum (Sigma) in phosphate-buffered saline containing 0.2% Triton X-100 (Sigma) for 1 h before incubation with primary antibodies in a humidified chamber at 37 °C overnight. The primary antibodies used (purchased from Abcam) included anti-OCT4 (1:200, Abcam, Cat. ab19857), anti-SOX2 (1:200, Abcam, Cat. ab97959), anti-TRA-1-60 (1:200, Abcam, Cat. ab16288), anti-SSEA4 (1:200, Abcam, Cat. ab16287), anti-RHODOPSIN (1:300, Abcam, Cat. ab221664), anti-L/M OPSIN (1:300, Novus, Cat. NB110-74730), and anti-S-OPSIN (1:300, Novus, Cat. NBP2-58288). After being washed and incubated for 1 h at room temperature with secondary antibodies, the sections were counterstained with ProLong Gold with DAPI (Invitrogen, Cat. 62248) to reveal the cell nuclei. The images were obtained at the corresponding histologically defined sites of the sections using an Olympus FV3000 confocal microscope. All images in each individual experiment were acquired with a fixed detection gain. The images were processed and semiquantified via ImageJ.

### TdT-UTP nick end labeling staining

TdT-UTP nick end labeling (TUNEL) assays were performed with a one-step TUNEL kit according to the manufacturer's instructions (Beyotime, Shanghai, Cat. C1086). After deparaffinization and hydration of paraffin sections, add 20 μg/ml proteinase K (Beyotime, Shanghai, Cat. ST533) without DNase and incubate at room temperature for 30 min, then wash with PBS three times, 5 min each time. Add 50 μl of TUNEL detection reagent to the samples and incubate at 37 °C in the dark for 60 min. Afterward, add DAPI (Invitrogen, Cat. 62248) working solution and incubate for 5 min, then wash with PBS three times and rinse with ultrapure water once. Finally, apply an anti-fluorescence quenching mounting medium (Beyotime, Shanghai, Cat. P0126) to seal the slide. The FITC-labeled TUNEL-positive cells were imaged under a fluorescence microscope at 488 nm excitation and 530 nm emission wavelengths. The cells with green fluorescence were defined as apoptotic cells.

### Determination of apoptosis by flow cytometry

The number of apoptotic RO cells was quantified with an Annexin V-FITC detection kit (Beyotime, Cat. C1062L) according to the manufacturer's protocol. Briefly, all of the ROs were washed with 1 × phosphate-buffered saline (PBS) three times and digested with 10 mg/ml papain (Sigma. Cat. P4762) and 10 mg/ml collagenase IV (Sigma. Cat. C4-28) and collected by centrifugation (300 *g* ∗ 5min). After being washed with 1 × PBS three times, the cells were resuspended in 0.5 mL of Annexin binding buffer. After that, the cells were stained with binding buffer containing PI and Annexin-V-FITC for 15 minutes and finally subjected to flow cytometry (BD CANTO, USA).

### RNA-seq and transcriptomic analysis

ROs were collected before total RNA was extracted. RNA-seq analysis was performed on the Illumina NovaSeq 6000 platform at a depth of 60 million read pairs per sample. Three samples from each group (20 ROs per sample) were analyzed. The raw reads were aligned to the *Homo sapiens* genome (GRH38) via HISAT2 with default parameters. The HTSeq-count function of HTSeq was used to determine the number of aligned reads that fell under the exons of the gene (union of all exons of the gene) to present the expression of each gene. Differentially expressed genes (DEGs) were identified by using the DESeq2 package in the R environment. Finally, Gene Ontology (GO) analysis was performed.

### RNA extraction and quantitative reverse transcription PCR (RT-qPCR)

Total RNA was extracted from different groups of ROs with Trizol on ice (ThermoFisher, Cat. 15596018CN). cDNA reverse synthesis was performed using the Invitrogen SuperScript™ IV one step RT-PCR system (Thermo Fisher, Cat. 12595100) and Real time PCR were performed using SsoAdvanced^TM^ Universal SYBR® Green Supermix (Bio-Rad, Cat. 1725270) according to the manufacture's manual (Bio-Rad, USA). The real-time PCR program was performed as follows: 40 cycles of denaturation at 95 °C for 10 s, annealing for 30 s, and elongation at 50 °C–60 °C for 30s. Primers were listed below:

HMOX1: Forward Primer AAGACTGCGTTCCTGCTCAAC; Reverse Primer AAAGCCCTACAGCAACTGTCG.

NFE2L2: Forward Primer TCAGCGACGGAAAGAGTATGA; Reverse Primer CCACTGGTTTCTGACTGGATGT.

### Animals

Male 8-week-old C57BL/6J mice were provided by Beijing Vitalstar Biotechnology. All of the animals were housed in a pathogen-free, temperature-controlled animal facility with a 12/12-h light/dark cycle and were provided standard food and water ad libitum. All animal protocols were conducted according to the ARVO Statement for the Use of Animals in Ophthalmic and Vision Research. The animal protocol was approved by the Institutional Animal Care and Use Committee of the General Hospital of the Chinese People's Liberation Army and the Academy of Military Medical Sciences (ID number: 307-ky-090). All efforts were made to reduce the number of animals used and minimize the suffering caused by experimental procedures.

### Sodium iodate injury mouse model and sample collection

Sodium iodate was selected to establish a retinal degeneration model according to a previous publication.^26^ The mice were randomly divided into 8 groups (*n* = 6 for each group): control, control + metformin, NaIO_3_ injury, NaIO_3_ injury + metformin, NaIO_3_ injury + metformin pretreated for 7 days, and control + TN1, NaIO_3_ injury + TN1 and NaIO_3_ injury + TN1 pretreated for 7 days. The NaIO_3_-injured mice were administered a single dose of 35 mg/kg NaIO_3_ dissolved in saline via tail vein injection (100μl/mouse), whereas those in the control group were given an equivalent volume of saline. All of the NaIO_3_ injury and control mice were examined by electroretinography (ERG), and their eyeballs were collected on the 7th day by enucleating the mice and immersing the eyeballs in 4% paraformaldehyde in 0.1 M phosphate buffer.

### HE staining and panretinal analysis

After 24 h of fixation, the anterior sections of the eyes were dissected, and the eye cups were dehydrated and embedded in paraffin wax. Sections (5 μm thick) were cut on a microtome (Shandon AS325; Thermo Scientific, United States). All histologic analyses of outer nuclear layer (ONL) thickness were performed using retinal sections cut along the parasagittal plane (super inferior). These sections also included the ocular nerve head to maintain regional consistency among replicates. H&E staining was then performed, and images were captured with a microscope (Olympus, Japan). For each section, 18 sites spaced 200 μm apart were used to analyze the outer nuclear layer (ONL) thickness. Three measurements were made per sample and averaged.

### ERG assay

ERG was performed using the Espion E3 console in conjunction with ColorDome (Diagnosys LLC). ERG experiments were carried out one week after injection. The mice were dark-adapted the night before the recordings were performed. They were anesthetized via a subcutaneous injection of xylazine (15 mg/kg) and ketamine (110 mg/kg). The pupils of the mice were dilated with 1% tropicamide, while the mice were positioned on a water warming pad to prevent hypothermia. For each mouse, only the right eye was examined. Active gold electrodes were placed on the right eye cornea as the recording electrodes. The reference and ground electrodes were placed subcutaneously in the mid-frontal areas of the head and tail, respectively. Light stimulation was applied at a density of 0.5 log (cd⋅s/m^2^) (scotopic condition). The amplitudes of the a- and b-waves were recorded and processed using a RETI-Port device (Roland Consult). All procedures were performed in a dark room under a dim red safety light.

### Statistical analysis

All of the data are presented as the means ± SDs. Normality was assessed by the Shapiro Wilk test. For data meeting the assumption of normality, and comparisons among multiple groups were determined by One-way analysis of variance (ANOVA), followed by Tukey's test. *p* < 0.05 was considered statistically significant.

## Results

### Development of human AMD retinal organoids

**Figure 1:**
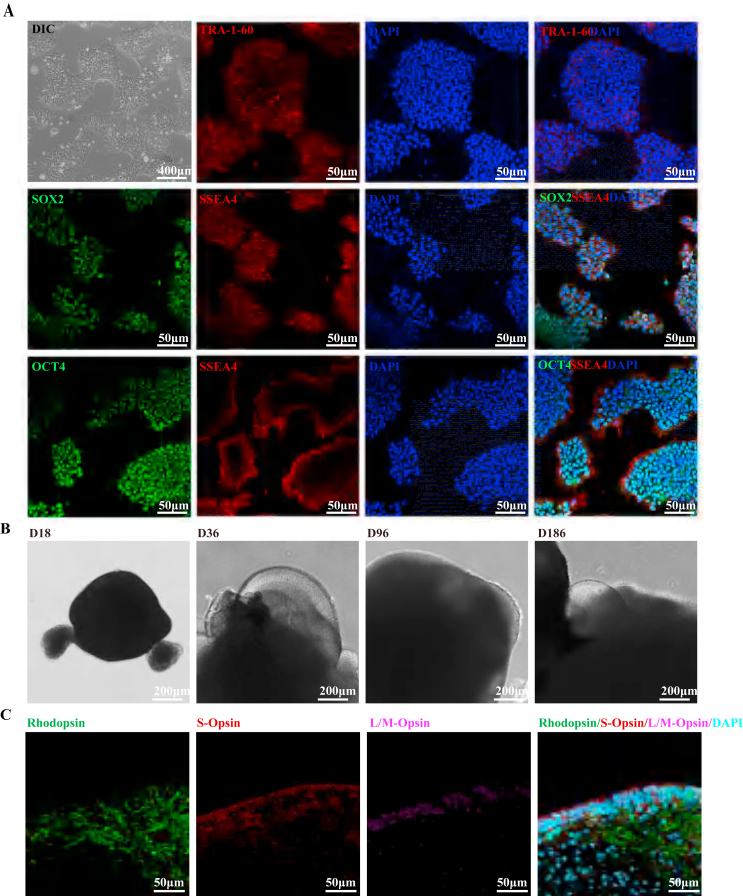
Generation of 3D retinal organoids from hESCs. **(A)** Bright-field images and repressive immunofluorescence images showing that hESCs formed classical colonies and highly expressed SOX2/OCT4/TRA-1-60/SSEA4. **(B)** Bright-field images showing the appearance of organoid cultures at different stages from day 18 to day 186. **(C)** Representative examples from day 186 ROs showing the expression of the photoreceptor markers RHODOPSIN/S–OPSIN/L/M-OPSIN.

The hESC H9 cell line was used to conduct 3D differentiation of ROs. After the hESC H9 cells were resuscitated and passaged 6 times, the cells formed uniform clones under differential interference contrast (DIC) microscopy. Furthermore, immunofluorescence staining revealed that all of the cells uniformly expressed high levels of the core characteristic transcription factor OCT4/SOX2/TRA-1-60/SSEA4 (Fig. 1A). These results confirmed that the H9 cells were in good condition, so 3D differentiation of ROs was started according to a previous protocol.^19^ DIC microscopy was used to observe the ROs, which were found to be composed mainly of neuroretinal and pigment parts. With differentiation, the volume of the ROs gradually increased. At the late stage of differentiation, cilia around the neuroretinal area were observed at approximately the 186th day (Fig. 1B). A previous single-cell transcriptome sequencing analysis revealed that ROs on the 186th day contained a large number of photoreceptor cells (PCs).^19^ Multi-immunofluorescence staining was used to assay the expression levels of photoreceptor cell-specific genes. Confocal microscopy revealed that the photoreceptor marker genes rhodopsin, S-Opsin and L/M-Opsin were highly expressed in neural-RO sections 186 days after initiation of 3D differentiation. The PCs expressing the three types of opsins were distributed mainly in the peripheral region (Fig. 1C). The above results and those of the previous single-cell transcriptome sequencing analysis suggested that ROs could be used 186 days after differentiation to construct a dry AMD model.

**Figure 2:**
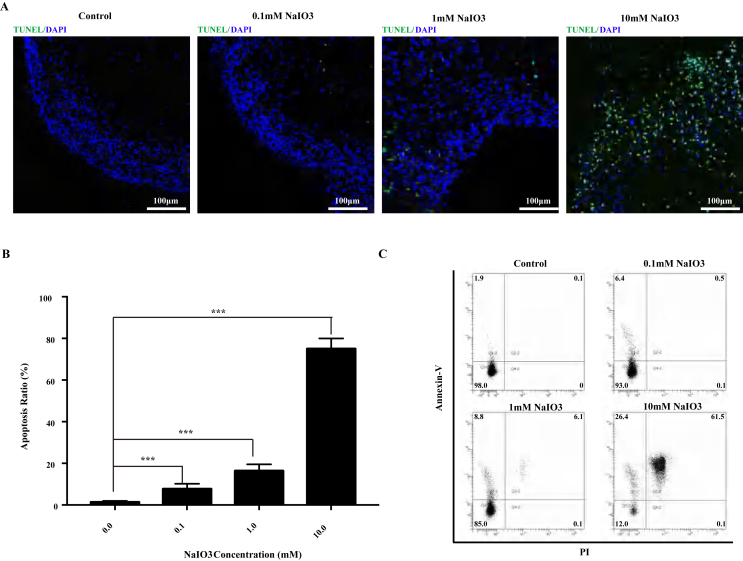
Development of the dry AMD RO model. **(A)** Bright-field images of human ROs at D7 after the application of different concentrations of sodium iodate (0, 0.1, 1 and 10 mM). **(B)** Representative confocal images of TUNEL apoptosis staining of sections from the control and NaIO_3_ injury groups of ROs on day 7 showing apoptotic cells after the application of sodium iodate. **(C)** The percentage of apoptotic cells increased with increasing sodium iodate concentration. (*n* = 6/group). (Data are shown as the mean ± SD. Statistical analyses are determined by One-way ANOVA, followed by Tukey's test. ∗∗∗*P* < 0.001).

In many previous animal experiments, sodium iodate was injected via the tail vein to simulate AMD.^27^, ^28^, ^29^ Therefore, to establish an hRO-AMD model, different dosages of sodium iodate were added to the RO culture medium and morphological changes in the ROs were observed. We divided the samples into four groups: normal control, 0.1 mM sodium iodate, 1 mM sodium iodate, and 10 mM sodium iodate. Compared with the normal control group, there was no looseness of RO tissue 7 days after 0.1 mM, 1 mM or 10 mM sodium iodate was added (Fig. 2A). Furthermore, the ROs were fixed and sectioned or digested for flow cytometry analysis 7 days after stimulation with sodium iodate. TUNEL immunofluorescence staining revealed that there were no TUNEL-positive cells in the ROs of the normal control group and few TUNEL-positive cells in the ROs of the 0.1 mM sodium iodate group (Fig. 2A, B). With increasing sodium iodate dosage, the proportion of TUNEL-positive cells in the ROs increased to 76% ± 6% in the 10 mM sodium iodate group (Fig. 2B), and the flow cytometry data revealed a similar trend (Fig. 2C). These results suggest that sodium iodate was able to induce oxidative stress injury in ROs in a dose-dependent manner. Therefore, we used 10 mM sodium iodate in the RO injury experiments.

### Transcriptomic analysis of human AMD retinal organoids

**Figure 3:**
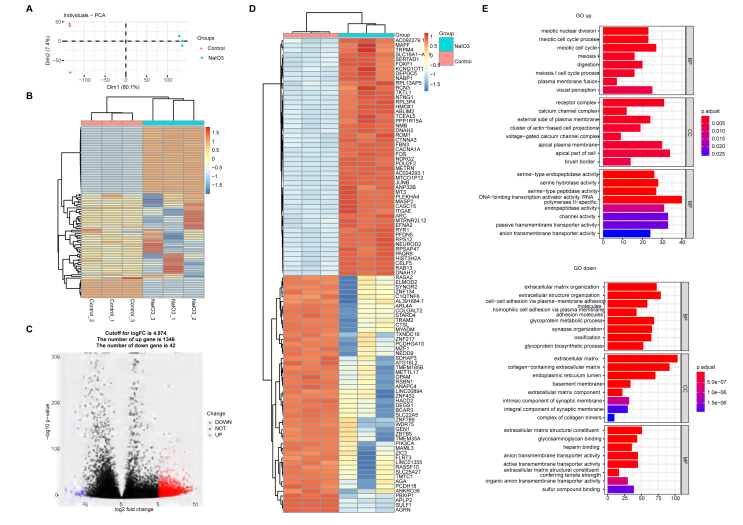
Transcriptomic analysis of the dry AMD RO model. **(A)** PCA graph showing the distribution of the control and NaIO_3_ injury groups. **(B)** Unsupervised clustering of the top 1000 genes expressed above background levels demonstrating similar patterns of gene expression between the control and NaIO_3_ injury groups (*n* = 3/group). **(C)** Volcano plots highlighting the distributions of DEGs between the control and NaIO_3_ injury groups. **(D)** Heatmap of the top 50 differentially expressed complement component genes between the control and NaIO_3_ injury groups. **(E)** Gene Ontology analysis of the signaling pathways related to up- and downregulated genes in the control and NaIO_3_ injury groups.

To determine the effect of sodium iodate on the gene expression of human ROs, organoid samples were selected from the normal control and 10 mM sodium iodate injury groups for transcriptome sequencing. As shown in the PCA map and gene expression heatmap, high repeatability of the three samples in each group was observed (Fig. 3A). Sodium iodate caused significant changes in the transcriptome properties of ROs. Compared with those in the normal control group, 1388 differentially expressed (DE) genes were induced by 10 mM sodium iodate stimulation and are displayed on a volcano map, 1346 of which were upregulated, whereas 42 were downregulated (Fig. 3C). The top 50 upregulated genes and the 42 downregulated genes were selected and are shown in a heatmap (Fig. 3D). Gene Ontology (GO) analysis indicated that the upregulated genes were involved in meiotic cell cycle processes, receptor complexes, and serine-type endopeptidase activities, whereas the downregulated genes were involved in extracellular matrix organization, the extracellular matrix and glycosaminoglycan binding after the use of sodium iodate (Fig. 3E). Furthermore, DE genes related to apoptosis and oxidative stress, retinal pigment epithelium (RPE)-related genes and PC-related genes were extracted. Compared with the normal control group, the expression of apoptosis-related genes, such as CASPASE10, increased in the sodium iodate group. The DUOX2, NUDT1, NOX5, HMOX1 and DUOX1 genes were upregulated, whereas the PRNP, DHCR24, PXDN and SOD3 genes were downregulated. The downregulated TTR, BEST1, PMEL and TYR genes were related to normal RPE function, whereas the downregulated CNGB3, PALMD, ABCA1 and SLC6A1 genes were associated with PC function (Fig. S1). These data indicated that sodium iodate could induce oxidative stress in human ROs and injury to the RPE and PCs, as evidenced by a sodium iodate-induced mouse degeneration model. The above results support the use of sodium-injured human ROs to model human AMD.

### Metformin rescued human AMD retinal organoid and mouse retinal injury

**Figure 4:**
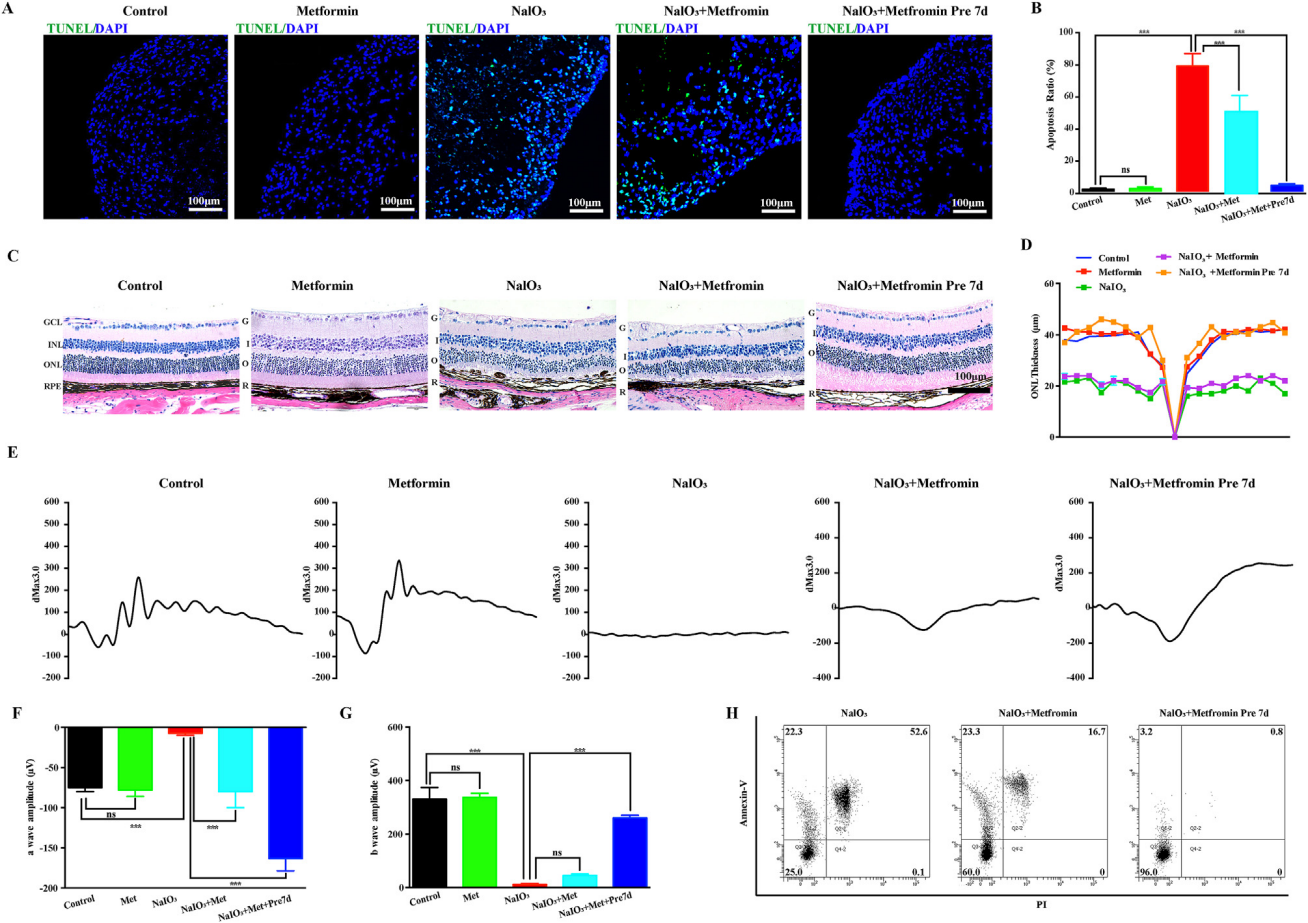
Metformin rescued RO and mouse retinal function after NaIO_3_ induction. **(A)** TUNEL assay of control, control + metformin, NaIO_3_ injury, NaIO_3_ injury + metformin and NaIO_3_ injury + metformin organoids 7 day pretreatment groups. No TUNEL-positive cells (green) were observed in the control, control + metformin or NaIO_3_ injury + metformin 7 day pretreatment groups. Many TUNEL-positive cells were observed in the NaIO_3_ injury group, whereas few TUNEL-positive cells were observed in the NaIO_3_ injury + metformin group. **(B)** Quantification of TUNEL in the control, control + metformin, NaIO_3_ injury, NaIO_3_ injury + metformin and NaIO_3_ injury + metformin RO 7 day pretreatment groups (*n* = 6/group). **(C)** H&E staining of retinas from the control, control + metformin, NaIO_3_ injury, NaIO_3_ injury + metformin and NaIO_3_ injury + metformin 7 day pretreatment groups. **(D)** Quantification of ONL thickness in the retinas of the control, control + metformin, NaIO_3_ injury, NaIO_3_ injury + metformin and NaIO_3_ injury + metformin 7 day pretreatment groups (*n* = 6/group). **(E)** ERG analysis of retinal function in the control, control + metformin, NaIO_3_ injury, NaIO_3_ injury + metformin and NaIO_3_ injury + metformin 7 day pretreatment groups. **(F, G)** Bar plot of the amplitudes of a and b waves in the control, control + metformin, NaIO_3_ injury, NaIO_3_ injury + metformin and NaIO_3_ injury + metformin 7 day pretreatment groups (*n* = 6/group). **(H)** Flow cytometry analysis of retinal apoptosis in the NaIO_3_ injury, NaIO_3_ injury + metformin and NaIO_3_ injury + metformin 7 day pretreatment groups. (*n* = 6/group). (Data are shown as the mean ± SD. Statistical analyses are determined by One-way ANOVA, followed by Tukey's test. ∗∗∗*P* < 0.001).

To date, there are very few medicines for AMD, so the hRO-AMD model we have established can be used to test new prospective drugs. Previous studies strongly suggested a protective effect of metformin on photoreceptor cells and RPEs, but relevant data in humans are still lacking.^30^ Therefore, we used this model to test the protective effect of metformin. TUNEL immunofluorescence staining revealed that metformin (100 μM) stimulation did not cause apoptosis in ROs, confirming its safety. Metformin significantly reduced the apoptosis of ROs induced by sodium iodate, especially in the group pretreated with metformin for 7 days, and the flow cytometry data were highly consistent with the immunofluorescence staining data (Fig. 4 A, B, H). In our study, sodium iodate was used to induce damage to the retinas of mice to verify the conclusions drawn from the experiments on human ROs. The hematoxylin-eosin (HE) staining results revealed that metformin pretreatment attenuated the structural disorders of the photoreceptor cell layer and RPE cell layer induced by sodium iodate (Fig. 4C and D). The electroretinography (ERG) results revealed that metformin protected against the decrease in a- and b-waves in the mice and significantly restored the amplitudes of these waves (Fig. 4E, F, G).

**Figure 5:**
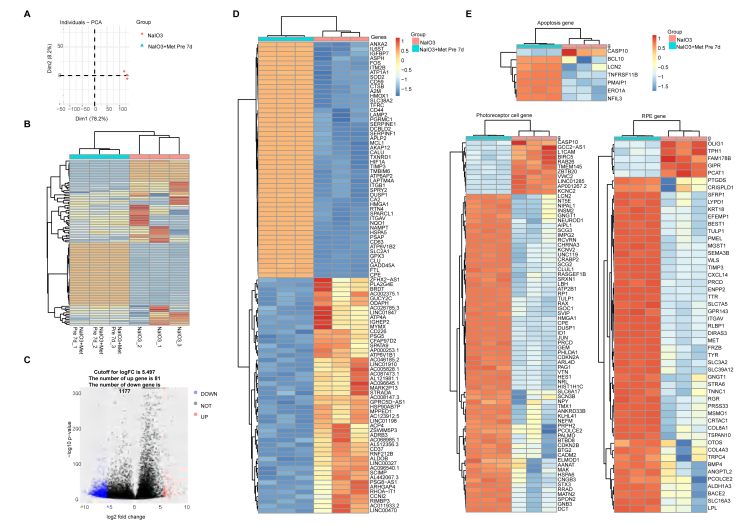
Transcriptomic analysis of metformin in the dry AMD RO model. **(A)** PCA graph showing the distribution of NaIO_3_ injury and NaIO_3_ injury + metformin 7 day pretreatment groups. **(B)** Unsupervised clustering of the top 1000 genes expressed above background levels revealed similar patterns of gene expression between the NaIO_3_ injury and NaIO_3_ injury + metformin 7 day pretreatment groups. **(C)** Volcano plots highlighting the distributions of DEGs between the NaIO_3_ injury and NaIO_3_ injury + metformin 7 day pretreatment groups. **(D)** Heatmap showing the top 50 differentially expressed complement component genes between the NaIO_3_ injury and NaIO_3_ injury + metformin 7 day pretreatment groups. **(E)** Heatmap showing the apoptosis-associated, photoreceptor cell-related and RPE-related differentially expressed genes between the NaIO_3_ injury and NaIO_3_ injury + metformin 7 day pretreatment groups.

To elucidate the molecular mechanism by which metformin protects human ROs from oxidative stress injury, samples were collected from the sodium iodate injury group and the sodium iodate injury plus metformin pretreatment group for RNA sequencing. The PCA and gene expression heatmap indicated the homogeneity of these samples, which could be divided into two groups, as in the biological grouping (Fig. 5A, B). Differential gene expression analysis identified 1258 differentially expressed genes. Compared with those in the sodium iodate injury group, 81 genes were upregulated and 1177 genes were downregulated after metformin treatment (Fig. 5C). The top 50 genes were selected for heatmap display (Fig. 5D). The expression levels of genes related to antioxidant stress, such as SOD2, HMOX1, HIF1A and NQO1, were significantly increased. Moreover, the expression of the CASPASE10 gene related to apoptosis was significantly reduced, and the expression of genes related to PCs, such as GNGT1, AIPL1, RCVRN and CLUL1, was significantly restored, as was the expression of genes related to RPE, such as BEST1, PMEL, TTR, MET and TYR (Fig. 5E). These results indicate that metformin can significantly alleviate the oxidative stress response of human ROs induced by sodium iodate and reduce damage to photoreceptors and RPE cells.

### TN1 relieved human AMD retinal organoid and mouse retinal injury

**Figure 6:**
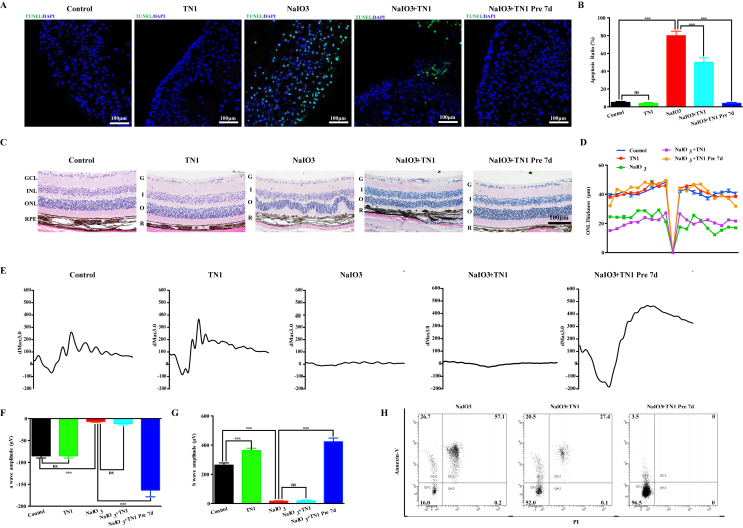
TN1 rescued RO and mouse retinal function after NaIO_3_ induction. **(A)** TUNEL assay of control, control + TN1, NaIO_3_ injury, NaIO_3_ injury + TN1 and NaIO_3_ injury + TN1 ROs pretreated for 7 days. No TUNEL-positive cells (green) were observed in the control, control + TN1 or NaIO_3_ injury + TN1 7 day pretreatment groups. Many TUNEL-positive cells were observed in the NaIO_3_ injury group, whereas few TUNEL-positive cells were observed in the NaIO_3_ injury + TN1 group. **(B)** Quantification of TUNEL in the control, control + TN1, NaIO_3_ injury, NaIO_3_ injury + TN1 and NaIO_3_ injury + TN1 7 day pretreatment groups of ROs (*n* = 6/group). **(C)** H&E staining of the retinas of the control, control + TN1, NaIO_3_ injury, NaIO_3_ injury + TN1 and NaIO_3_ injury + TN1 7 day pretreatment groups of mice. **(D)** Quantification of ONL thickness in the retinas of control, control + TN1, NaIO_3_ injury, NaIO_3_ injury + TN1 and NaIO_3_ injury + TN1 7 day pretreatment groups of mice (*n* = 6/group). **(E)** ERG analysis of retinal function in the control, control + TN1, NaIO_3_ injury, NaIO_3_ injury + TN1 and NaIO_3_ injury + TN1 7 day pretreatment groups of mice. **(F, G)** Bar plot of the amplitudes of a and b waves in the control, control + TN1, NaIO_3_ injury, NaIO_3_ injury + TN1 and NaIO_3_ injury + TN1 7 day pretreatment groups of mice. ∗∗∗*P* < 0.001 (*n* = 6/group). **(H)** Flow cytometry analysis of retinal apoptosis in NaIO_3_ injury, NaIO_3_ injury + TN1 and NaIO_3_ injury + TN1 7 day pretreatment groups (*n* = 6/group). (Data are shown as the mean ± SD. Statistical analyses are determined by One-way ANOVA, followed by Tukey's test. ∗∗∗*P* < 0.001).

**Figure 7:**
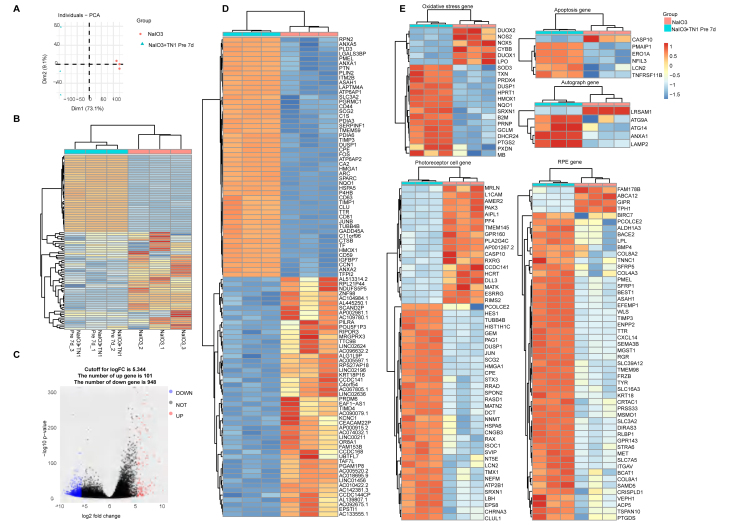
Transcriptomic analysis of the effect of TN1 on the human dry AMD retinal organoid model. **(A)** PCA graph showing the distributions of the NaIO_3_ injury and NaIO_3_ injury + TN1 7 day pretreatment groups (*n* = 3/group). **(B)** Unsupervised clustering of the top 1000 genes expressed above background levels revealing similar patterns of gene expression between the NaIO_3_ injury and NaIO_3_ injury + TN1 7 day pretreatment groups. **(C)** Volcano plots highlighting the distributions of DEGs between the NaIO_3_ injury group and the NaIO_3_+TN1 7 day pretreatment groups. **(D)** Heatmap of the top 50 differentially expressed complement component genes between the NaIO_3_ injury group and the NaIO_3_+TN1 7 day pretreatment groups. **(E)** Heatmap of apoptosis-associated, photoreceptor cell-related and RPE-related differentially expressed genes between the NaIO_3_ injury group and the NaIO_3_+ TN1 7 day pretreatment groups.

Previous studies have suggested that activating HbF can rescue retinal degeneration caused by anemia and other related diseases.^31^^,^^32^ However, it is unclear whether activating HbF has a protective effect on AMD. Next, we used this AMD model to test the protective effect of the fetal hemoglobin agonist TN1. TUNEL immunofluorescence staining revealed that compared with the control treatment, TN1 treatment did not cause apoptosis in ROs. The addition of TN1 during the course of sodium iodate injury reduced cell apoptosis to some extent, but treatment with TN1 7 days before sodium iodate injury significantly mitigated apoptosis, and the flow cytometry data were highly consistent with the results of the immunofluorescence staining (Fig. 6A, B, H), suggesting that TN1 effectively alleviated the apoptosis of ROs caused by oxidative stress induced by sodium iodate. The above results were verified in a mouse model of retinal injury and degeneration induced by sodium iodate. The addition of TN1 at the onset of sodium iodate injury alleviated retinal degeneration. The retinal structure of the mice injected with TN1 7 days before sodium iodate injury induction was not significantly different from that of the control group, suggesting that TN1 could protect against damage to the retina caused by sodium iodate (Fig. 6C, D). The ERG results revealed that the a- and b-waves of the ERG waveform completely disappeared 7 days after sodium iodate injury, indicating that visual function was severely impaired. Weak residual b-waves were detected in the NaIO_3_ injury + TN1 groups, but the amplitudes of the a- and b-waves in the NaIO_3_ injury + TN1 7 days’ pretreatment group did not significantly decrease compared with those in the normal group (Fig. 6E, F, G). These results also confirmed that TN1 could protect the retina from oxidative stress injury and was highly applicable. However, the molecular mechanism by which TN1 protects human ROs remains unclear. We subsequently collected RO samples from the NaIO_3_ injury group and the NaIO_3_ injury + TN1 7 day pretreatment group for RNA sequencing analysis. The PCA and gene expression heatmap revealed that the samples could be divided into two groups according to their biological grouping (Fig. 7A, B). Differential gene expression analysis revealed that, compared with the NaIO_3_ injury group, the TN1 treatment group presented changes in the expression of 1049 genes. As shown in the volcano map, 101 genes were upregulated, whereas 948 were downregulated (the cutoff for log2FC was 5.344) (Fig. 7C). Subsequent differential gene analysis revealed that TN1 treatment led to a significant increase in the expression of SOD3, HMOX1, NQO1 and GCLM, which are involved in antioxidant stress; a significant decrease in the expression of the apoptosis-related gene CASPASE10, and an increase in the expression of the antiapoptosis genes PMAIP1 and LCN2. TN1 treatment protected against the upregulation of the photoreceptor function-related genes PAG1, SCG2, and CLUL1 and the RPE-related genes PMEL, BEST1, TMEM98, TTR, and MET (Fig. 7D, E). These data indicate that TN1 can significantly alleviate the oxidative stress response of human ROs induced by sodium iodate and reduce functional damage to photoreceptors and RPE cells.

### Transcriptomic characteristics of metformin and TN1 in human retinal organoids

Metformin and TN1 protected ROs against oxidative stress induced by sodium iodate. Pretreatment with these two drugs increased the resistance of human ROs to oxidative stress. To explore the mechanism of protection, the changes in molecular expression after metformin and TN1 treatment of normal ROs were analyzed. With RNA-seq sequencing, DEG2 was used to identify overlapping differentially expressed genes. Both drugs upregulated HMOX1 (Fig. S2, 3). Furthermore, we applied real-time PCR to detect the expression levels of NFE2L2/HMOX1. The results showed that after sodium iodate injury, the mRNA level of NFE2L2 in ROs significantly dropped, while the mRNA level of HMOX1 slightly increased. The use of metformin cannot alter the NFE2L2 mRNA level in normal ROs, but it can increase the mRNA level of HMOX1, especially in ROs damaged by sodium iodate. At the same time or pre-treatment with metformin in sodium iodate injury group, the NFE2L2 mRNA level significantly return to near normal levels, and the HMOX1 mRNA level increase significantly. We also found that TN1 treatment did not reverse the decrease in NFE2L2 mRNA level caused by sodium iodate damage, but significantly increased HMOX1 mRNA level (Fig. S4). Taken together, these results suggest that HMOX1 may be an important molecule involved in the anti-oxidative stress injury of the two drugs mentioned above.

## Discussion

In this study, we developed a human retinal degeneration model by adding sodium iodate to the culture environment, which simulated the key pathological processes of human dry AMD. In addition, we identified two drugs with protective effects against retinal degeneration, metformin and the fetal hemoglobin inducer TN1, and found that the HMOX1 gene may be associated with this protective effect. These findings provide a molecular biological basis for the subsequent development of therapeutic treatments for dry AMD through the targeting of HMOX1 and its downstream pathways.

**Figure 8:**
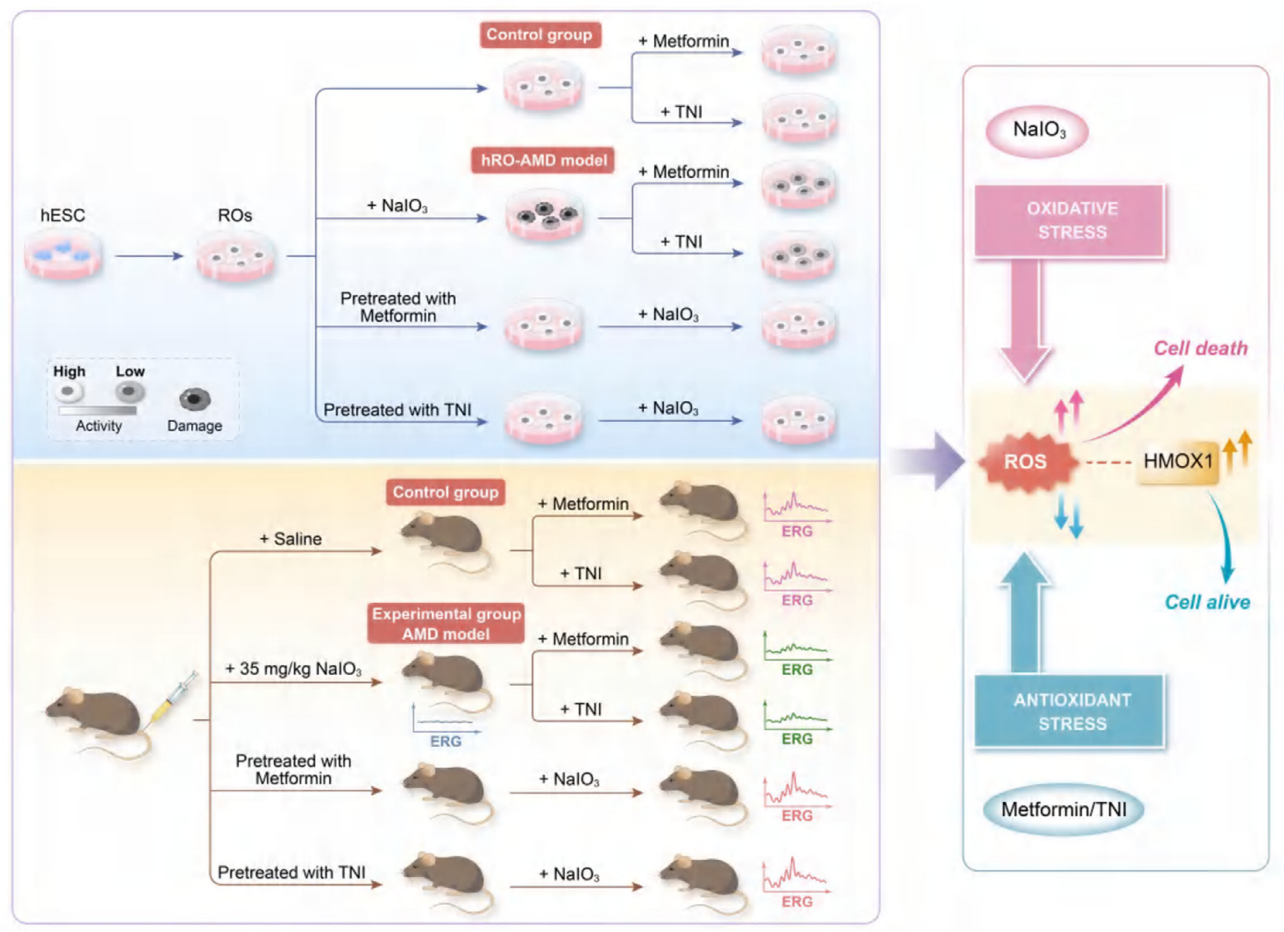
Schematic graph of the protective effects of metformin and fetal hemoglobin (HbF) inducer TN1 in sodium iodate-induced RO model and murine model.

Compared with animal models, human organoids provide a better platform for studying human diseases. In vitro human organoid models are becoming an increasingly popular research modality as a complement to animal models. Induced PSCs (iPSCs) retain an individual's specific genome and can differentiate into any type of adult cell. 3D iPSC-derived ROs contain all major retina-specific cell types. 3D organoid technology based on specific diseases can reveal clinical phenotypes that are remarkably similar to those of human patients, making it an ideal technology for modeling human retinal diseases. Although animal models have the advantages of low cost and ease of genetic manipulation, there are some differences in retinal anatomy and photoreceptor types compared with those of humans, and they are subject to ethical limitations. Many previous studies have used ROs to focus on the pathological retinal development caused by gene mutations. However, little research has been conducted on disease modeling through ROs for drug interventions. The sodium iodate-injured ROs established in this study constitute an hRO-AMD model for drug interventions. We began by establishing an in vitro oxidative stress degeneration model of human ROs via the use of various compounds. We subsequently explored the early molecular pathological processes of RO degeneration by performing many experiments to elucidate the molecular mechanism of dry AMD. These findings may provide potential tools for high-throughput drug screening and the discovery of new treatment methods and make accurate toxicity testing possible. These findings will deepen our understanding of the mechanisms of retinal degeneration and further increase the development of novel therapies^33^^,^^34^ (Fig. 8).

Next, we investigated the protective effects of metformin^30^ and the HbF inducer TN1 against retinal degeneration and the related molecular mechanisms by using this RO degeneration model. Previous studies have demonstrated that metformin can improve the ability of photoreceptors and RPE cells to resist oxidative stress, thus preventing DNA damage and oxidative stress damage in a murine model of retinal degeneration.^30^ However, due to the lack of appropriate models, the efficacy of metformin against human dry AMD and the possible molecular mechanism remain elusive, which is why the hRO-AMD model was used to explore the protective effect and molecular mechanism of metformin. In the hRO-AMD model, after metformin treatment, the levels of genes related to antioxidative stress were significantly increased, the expression of genes related to apoptosis was significantly decreased, and the expression of genes related to RPE and PCs was significantly restored, thus confirming that metformin significantly reduces sodium iodate-induced apoptosis in ROs, decreases oxidative stress in photoreceptors and alleviates RPE cell damage, overall providing a protective effect on the retina.

Studies have shown that, similar to red blood cells, retinal cells also express hemoglobin and that the activation of HbF can alleviate retinal degeneration retinopathy in a humanized mouse model of sickle cell disease.^31^^,^^32^ These results suggest the possibility of activating HbF for the treatment of retinal degeneration diseases, but there has been no related research thus far. In our study, after TN1 treatment, the expression of genes involved in anti-oxidative stress was significantly increased, the expression of apoptosis-related genes was significantly decreased, and the expression of anti-apoptotic genes was increased, suggesting that TN1 can significantly alleviate the damage caused by oxidative stress in ROs induced by sodium iodate. The protective effect of TN1 has also been validated in a mouse retinal injury model induced by sodium iodate, confirming the alleviating effect of TN1 on the hRO-AMD model and mouse retinal injury. Our study is likely the first to use TN1, a specific inducer of HbF, to treat hRO-AMD. The two drugs tested, which are based on the hRO-AMD model, have great potential for application, avoiding the possible failure associated with the use of experimental animals due to species differences. In addition to these two drugs, we can also test and screen other potential targets, such as those involved in cell death-related pathways and complement pathway-related targets. Undoubtedly, these findings will help us to better understand the mechanism of AMD and explore new biomolecules and therapeutic approaches.

In our study, RNA-seq analysis revealed a significant increase in the expression of the HMOX1 gene, which is involved in resistance to oxidative stress, after treatment with metformin or TN1 compared with that in the sodium iodate-injured group. Thus, it was hypothesized that the activation of the HMOX1 gene is a common pathway in the resistance to oxidative stress injury caused by both drugs, metformin and TN1. Previous studies have shown that HMOX1 is a classical regulator of the cellular pathway that protects against oxidative stress injury. Activation of the HMOX1 gene may play a dominant role in enhancing the ability of ROs to resist oxidative stress injury.^35^, ^36^, ^37^, ^38^ In our model, we observed slight upregulation of HMOX1 expression in ROs after treatment with sodium iodate injury, but this increase was not enough to counteract the damage caused by sodium iodate induction. However, the expression level of HMOX1 was significantly increased by the introduction of metformin and TN1 treatment. Therefore, we hypothesized that metformin and TN1 could activate the expression of HMOX1, thereby increasing the anti-oxidative stress capacity of human ROs. The above results suggest that insufficiently elevated HMOX1 expression may be the reason for the failure of cells to cope with oxidative stress injury; thus, agonists can be applied to increase HMOX1 expression in the later stage, or gene editing can be applied to intervene in the expression of HMOX1 to respond effectively to injury and reduce the risk of cell death. These results provide data supporting the future use of the hRO-AMD model to reveal the mechanisms involved in the regulation of HMOX1 expression during AMD pathogenesis, to screen small molecule compounds that target HMOX1 and its downstream pathway activation, and to explore new therapies for the treatment of human dry AMD.

One limitation of this study is the failure to simulate the entire pathogenesis of human dry AMD, including the lack of blood vessels and immune cells in ROs.^9^^,^^39^ Although the ROs used in this study already share the structure of photoreceptors, they are young-stage cells and are unlikely to simulate all of the changes that occur in old individuals. Slow maturation is another key factor limiting the modeling of disease in later developmental stages, so future attempts could be made to address this obstacle by pretreating organoids with small-molecule compounds.

In conclusion, the results from morphological, transcriptomic and electrophysiological assessments revealed that the oxidative stress injury model of ROs can simulate the oxidative stress injury processes of dry AMD in humans. Given the lack of a dry AMD human model, this oxidative stress injury model of ROs can serve as a good platform for accurately exploring intervention targets in dry AMD. This model can be used to directly test and predict potential pharmacological interventions for the dry AMD, which can greatly accelerating the development process of drugs.

## Copyright transfer agreement

The authors of this article (Title: Human Retinal Organoids for Modeling Dry Age-Related Macular Degeneration and Screening Drugs) agree to transfer the copyright to Elsevier.

## CRediT authorship contribution statement

**Xiaolu Hao:** Writing – review & editing, Writing – original draft, Visualization, Validation, Software, Methodology, Formal analysis, Data curation, Conceptualization. **Lu Du:** Writing – review & editing, Writing – original draft, Visualization, Validation, Software, Methodology, Formal analysis, Data curation, Conceptualization. **Guang Liu:** Writing – review & editing, Writing – original draft, Visualization, Validation, Supervision, Software, Resources, Project administration, Methodology, Investigation, Formal analysis, Data curation, Conceptualization. **Zhaohui Li:** Writing – review & editing, Writing – original draft, Visualization, Validation, Supervision, Resources, Project administration, Methodology, Investigation, Formal analysis. **Shaojun Wang:** Writing – review & editing, Writing – original draft, Validation, Supervision, Software, Resources, Project administration, Investigation, Funding acquisition, Data curation, Conceptualization.

## Conflict of interests

The authors declare that they have no competing interests.
