# Supplementary material for: Human retinal organoids for modelling dry age-related macular degeneration and screening drugs
Source: Genes Dis. 2025 Mar 7;12(6):101593. doi: 10.1016/j.gendis.2025.101593 (PMC12363467; doi:10.1016/j.gendis.2025.101593)
Supplement: Multimedia component 1 [file mmc1.docx]

**Figure S1** Transcriptomic analysis of apoptosis and oxidative stress in the retinal organoid model of dry age-related macular degeneration. The heatmaps showed differences in the expression of genes related to apoptosis and oxidative stress, photoreceptor cell-related genes, and retinal pigment epithelial cell-related genes between the control and NaIO_3_ injury groups.

**Figure S2** Transcriptomic analysis of the effect of metformin on normal retinal organoids. **(A)** Principal component analysis plot showing the distribution of the control and control+metformin groups (*n* = 3/group). **(B)** Unsupervised clustering of the top 1000 genes expressed above background levels showing similar patterns of gene expression between the control and control+metformin groups. **(C)** Volcano plots highlighting the distributions of the differentially expressed genes between the control and control+metformin groups. **(D)** Heatmap of the top 50 differentially expressed complement component genes between the control and control+metformin groups. **(E)** Heatmap of the differentially expressed photoreceptor cell-related and retinal pigment epithelial cell-related genes between the control and control+metformin groups. **(F)** Gene ontology analysis of the signaling pathways related to the up- and down-regulated genes in the control and control+metformin groups.

**Figure S3** Transcriptomic analysis of the effect of TN1 on normal retinal organoids. **(A)** Principal component analysis plot showing the distribution of the control and control+TN1 groups (*n* = 3/group). **(B)** Unsupervised clustering of the top 1000 genes expressed above background levels showing similar patterns of gene expression between the control and control+TN1 groups. **(C)** Volcano plots highlighting the distributions of differentially expressed genes between the control and control+TN1 groups. **(D)** Heatmap of the top 50 differentially expressed complement component genes between the control and control+TN1 groups. **(E)** Heatmap of the differentially expressed photoreceptor cell-related and retinal pigment epithelial cell-related genes between the control and control+TN1 groups. **(F)** Gene ontology analysis of the signaling pathways related to up- and down-regulated genes in the control and control+TN1 groups.

**Figure S4** Real-time PCR assay of NEF2L2 and HOMX1 gene expression. **(A, B)** Real-time PCR of NEF2L2 and HOMX1 gene expression in the retinal organoids of the control, control+metformin, NaIO_3_ injury, NaIO_3_ injury+metformin, and NaIO_3_ injury+metformin 7-day pretreatment groups (*n* = 3/group). **(C, D)** Real-time PCR of NEF2L2 and HOMX1 gene expression in the retinal organoids of the control, control+TN1, NaIO_3_ injury, NaIO_3_ injury+TN1, and NaIO_3_ injury+TN1 7-day pretreatment groups. (*n* = 3/group). Data were shown as mean ± standard deviation. Statistical analyses were determined by one-way ANOVA, followed by Tukey's test. ^***^*P* < 0.001.
