# Supplementary figures and images for: Human retinal organoids for modelling dry age-related macular degeneration and screening drugs
Source: Genes Dis. 2025 Mar 7;12(6):101593. doi: 10.1016/j.gendis.2025.101593 (PMC12363467; doi:10.1016/j.gendis.2025.101593)

Figure S1

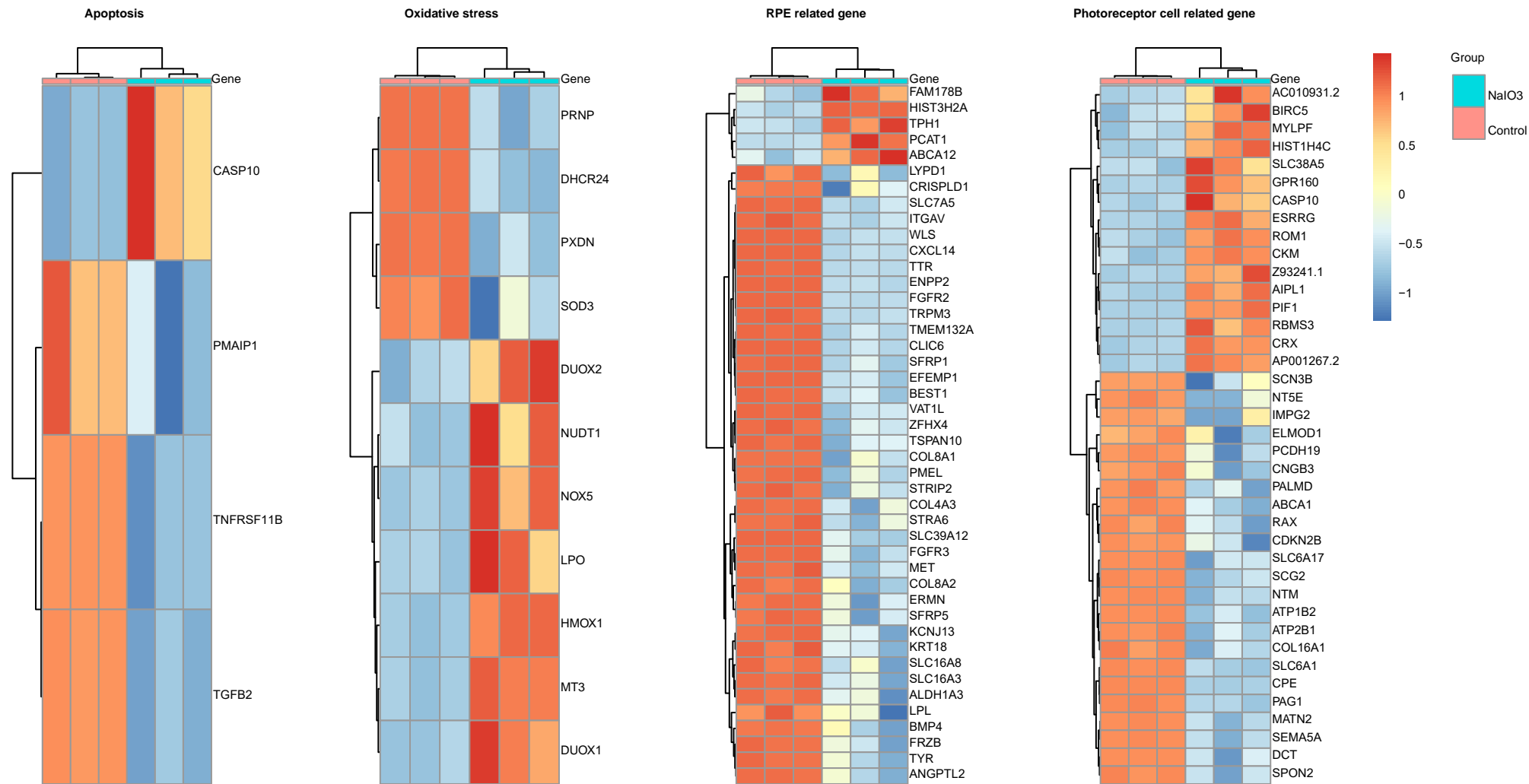

Supplement: Multimedia component 2 [file mmc2.pdf]

Figure S2

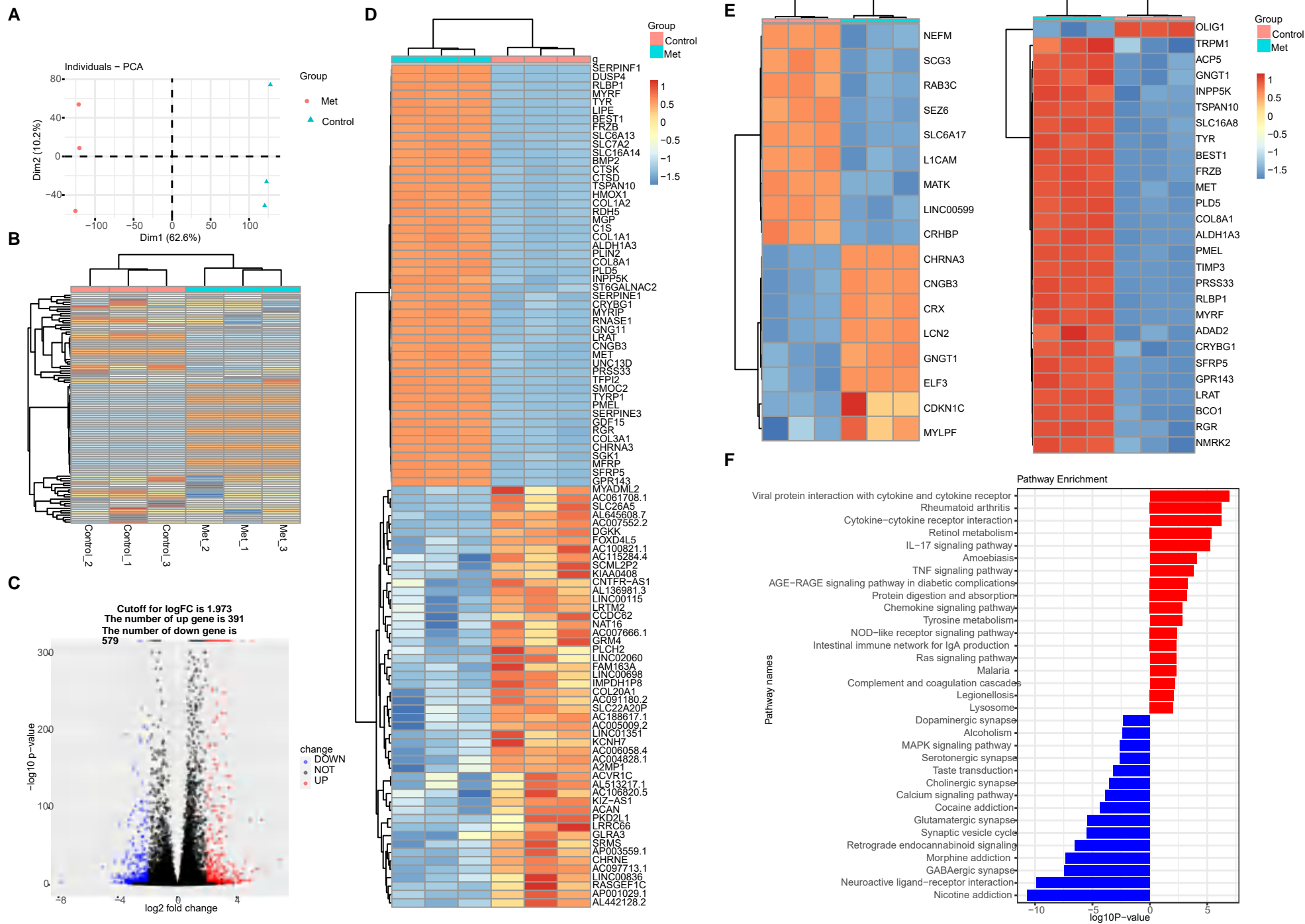

Supplement: Multimedia component 3 [file mmc3.pdf]

Figure S3

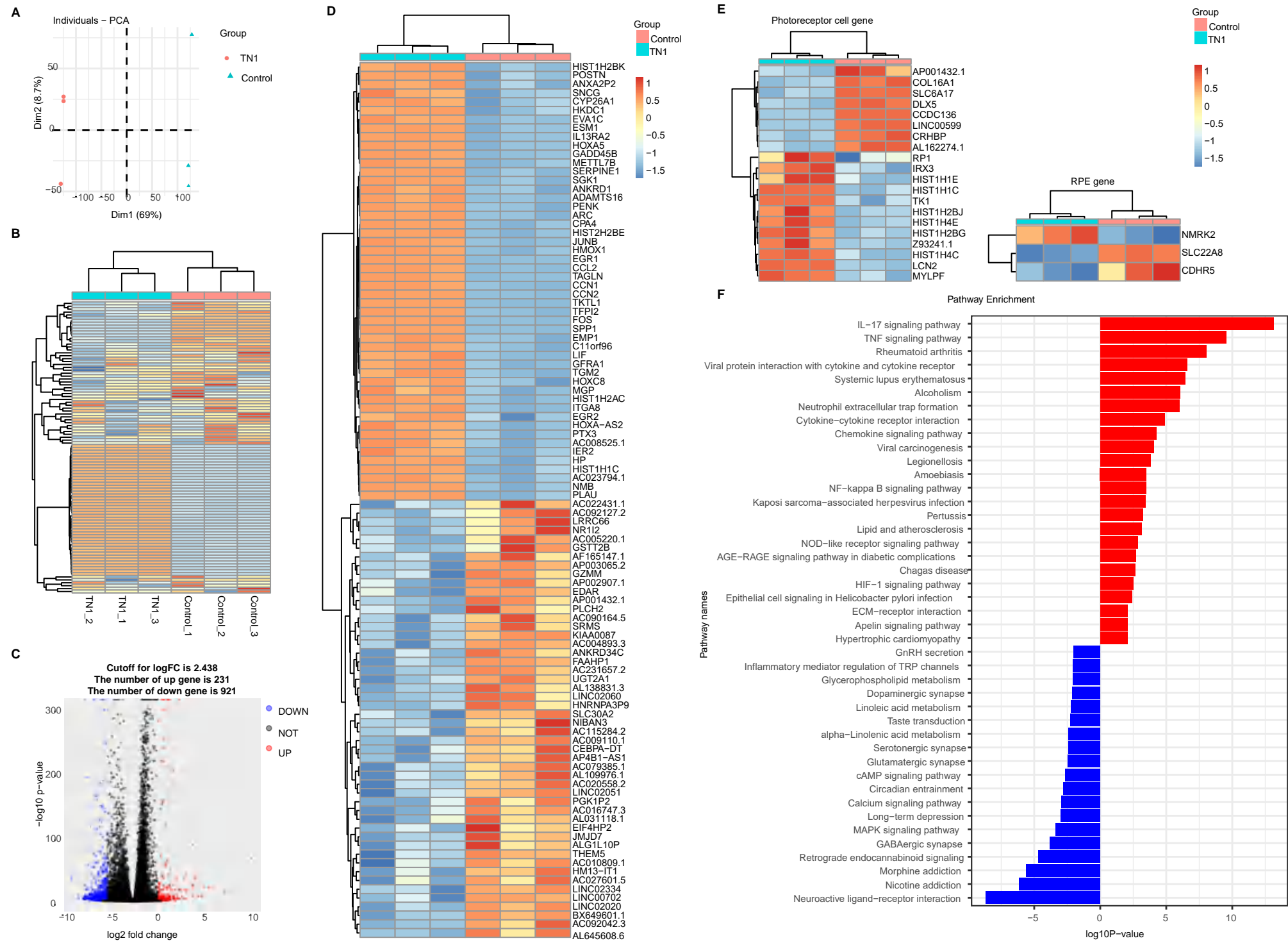

Supplement: Multimedia component 4 [file mmc4.pdf]

A

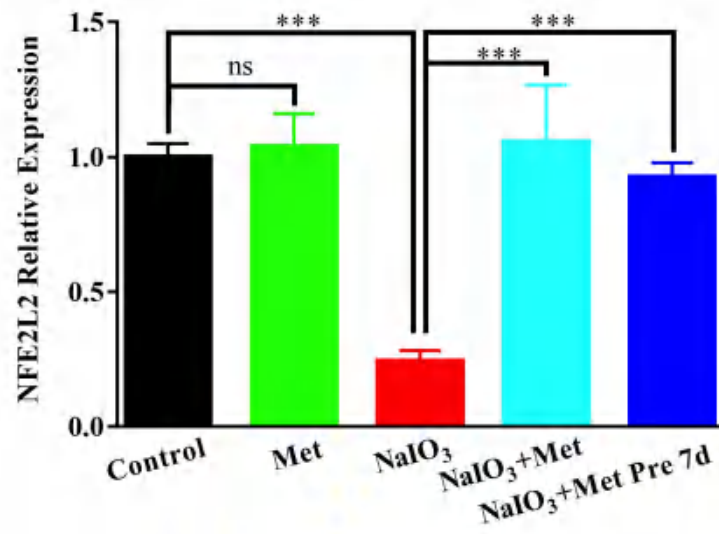

B

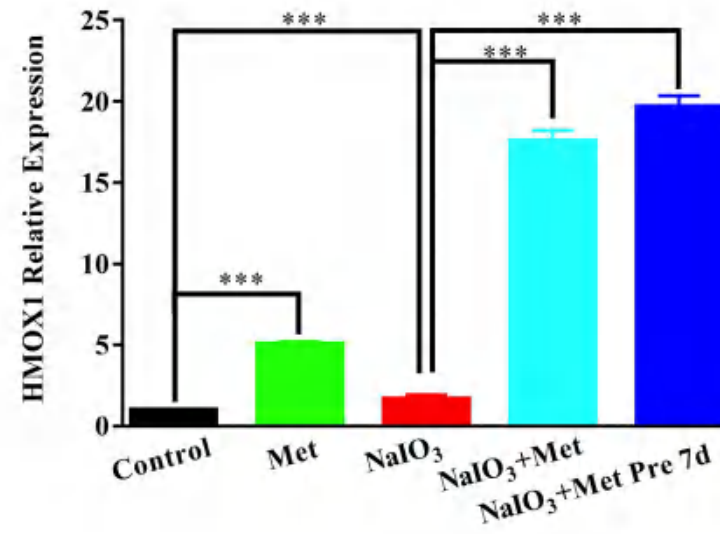

C

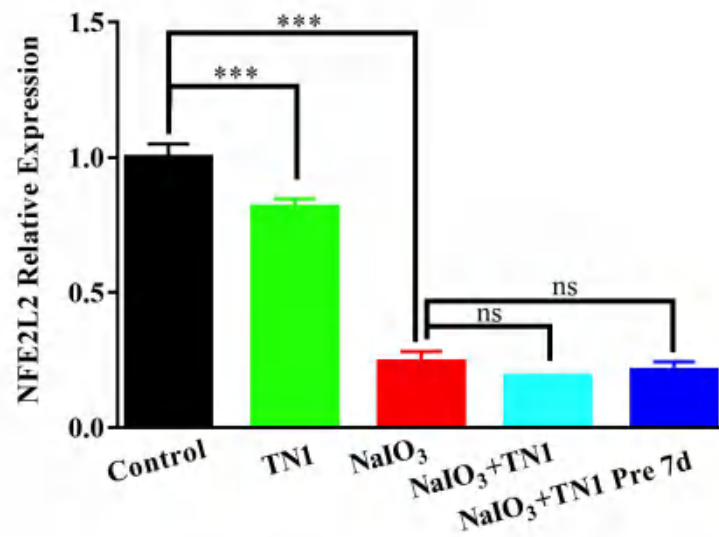

D

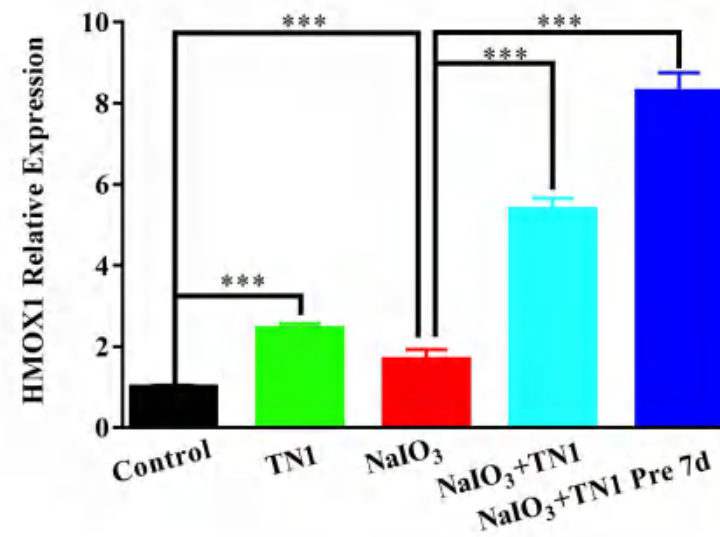

Supplement: Multimedia component 5 [file mmc5.pdf]
